# Dynamic plant spacing in tomato results in high yields while mitigating the reduction in fruit quality associated with high planting densities

**DOI:** 10.3389/fpls.2024.1386950

**Published:** 2024-04-18

**Authors:** Margarethe Karpe, Leo F. M. Marcelis, Ep Heuvelink

**Affiliations:** Horticulture and Product Physiology, Department of Plant Sciences, Wageningen University & Research, Wageningen, Netherlands

**Keywords:** light interception, planting density, dry matter partitioning, light use efficiency, dynamic spacing, dwarf tomato

## Abstract

High planting densities achieve high light interception and harvestable yield per area but at the expense of product quality. This study aimed to maintain high light interception without negative impacts on fruit quality. Dwarf tomato was grown at four densities in a climate-controlled room—at two constant densities (high and low) and two dynamic spacing treatments (maintaining 90% and 75% ground coverage by decreasing planting density in 3–4 steps)—resulting in ~100, 19, 54, and 41 plants/m^2^ averaged over 100 days of cultivation, respectively. Constant high density resulted in the highest light use efficiency (LUE; 7.7 g fruit fresh weight per mol photons incident on the canopy) and the highest harvestable fruit yield (11.1 kg/m^2^) but the lowest fruit size and quality. Constant low density resulted in the lowest LUE and yield (2.3 g/mol and 3.2 kg/m^2^, respectively), but higher fruit size and quality than high density. Compared to low density, maintaining 90% ground coverage increased yield (9.1 kg/m^2^) and LUE (6.4 g/mol). Maintaining 75% ground coverage resulted in a 7.2 kg/m^2^ yield and 5.1 g/mol LUE. Both dynamic spacing treatments attained the same or slightly reduced fruit quality compared to low density. Total plant weight per m^2^ increased with planting density and saturated at a constant high density. Assimilate shortage at the plant level and flower abortion lowered harvestable fruit yield per plant, sweetness, and acidity under constant high density. Harvestable fruit yield per plant was the highest under dynamic spacing and low density. Under constant high density, morphological responses to lower light availability per plant—i.e., higher specific leaf area, internode elongation, and increased slenderness—coincided with the improved whole-plant LUE (g plant dry weight per mol photons). We conclude that a constant high planting density results in the highest harvestable fruit yield per area, but with reduced fruit quality. Dynamic spacing during cultivation produces the same fruit quality as constant low density, but with more than double the harvestable yield per area.

## 1 Introduction

Agriculture aims to maximize yield and product quality to meet increasing consumer demands for nutritious food (Godfray et al., 2010). Optimizing planting density benefits both productivity per unit of cultivation area and product quality. At the start of cultivation, the total leaf area covering 1 m^2^ of cultivation area [leaf area index (LAI)] is low. Most of the incoming light is not intercepted by leaves and therefore lost for growth and yield formation. Consequently, growing small plants at high planting densities is favorable to increasing light capture, which drives subsequent physiological processes. Maximum light interception is usually reached at LAIs of three to four, with hardly any gain—but increasing competition for light—at higher LAIs (Heuvelink et al., 2004; Postma et al., 2021).

The degree of competition between plants continuously changes during growth and results in resource competition between individual plants at higher densities if resource availability is constant (Postma et al., 2021). Competition and resource limitations start once plants receive signals of neighboring plants’ proximity (Postma et al., 2021). Proximity (shading) signals from nearby vegetation include reduced light intensity, an increased red-to-far-red ratio (Franklin, 2008), and the touching of leaves (de Wit et al., 2012). Competition responses in dense canopies include limitations in biomass assimilation, changes in assimilate partitioning within the plant, and morphological adaptations to low-light environments (Franklin, 2008; Weiner and Freckleton, 2010; Postma et al., 2021). At high planting densities, plants are expected to have thinner leaves, thinner stems, more leaf senescence, and a lower reproductive effort (i.e., fruiting success; Postma et al., 2021). In tomatoes, high planting density—provided it is not so high that it prevents plants from producing enough assimilates to support generative growth—can hamper fruit set (Heuvelink, 1995; Amundson et al., 2012) and reduces fruit size and thus marketability, but it increases fruit yield per unit cultivation area (Cockshull and Ho, 1995).

In controlled environments where plants are commonly cultivated out of the soil, planting density can be managed through dynamic spacing to improve the efficiency of light and space. Especially in indoor plant production systems with solely artificial lighting (e.g., vertical farms), cultivation areas and electricity for light are expensive. Lighting was reported to constitute up to 80% of a vertical farm’s energy costs (Zeidler et al., 2017; Graamans et al., 2018; Kozai et al., 2019; Liu et al., 2023). If initially high planting densities can be reduced in several steps to maintain a constant degree of plant–plant competition while using available light efficiently, high productivity per cultivation area can be achieved, and fruit quality reductions can be avoided. Dynamic spacing during the cultivation of numerous crops, including dwarf tomato, is technically possible and viable with increasing automation. Few papers have been published on dynamic spacing (Leakey, 1971; Cockshull and Ho, 1995; Ioslovich and Gutman, 2000). Their applicability to commercial indoor production with artificial light spectra (which is often without far-red; Poorter et al., 2016) is limited mainly due to the presence of the solar light spectrum. Further limitations to implementing findings of those previous studies on indoor cultivation are 1) the choice of crop (barley in Leakey, 1971; high-wire tomato in Cockshull and Ho, 1995), 2) the applicability of the method to increase planting density during cultivation (resowing in Leakey, 1971; retention of additional high-wire tomato side shoots in Cockshull and Ho, 1995), and 3) the chosen mathematical modeling approach to determine optimal spacing in the absence of experimental validation (Ioslovich and Gutman, 2000).

We aimed to determine the effects of frequently decreasing planting density during cultivation on harvestable tomato fruit yield (per plant and per m^2^), light use efficiency (LUE; harvestable fruit yield per incident mol of photons), and consequences for fruit quality. We hypothesized that decreasing planting densities in tomato cultivation while maintaining a high ground coverage of 90% would outperform a constant high planting density due to trade-offs between fruit quality and harvestable yield under a constant high planting density. Constant high planting density was expected to result in morphological adaptations to low-light environments, such as undesired flower abortion and fewer, smaller fruits but also a more elongated, open architecture that benefits light interception throughout the canopy. A constant low planting density was hypothesized to intercept the lowest fraction of incident photons and to have the lowest harvestable yield per cultivation area and thus the lowest LUE. A fruit crop, the commercially available dwarf tomato cultivar “Plum Tomato Red”, was chosen as the experimental crop.

## 2 Materials and methods

### 2.1 Plant material and experimental setup

Dwarf tomato (*Solanum lycopersicum* “Plum Tomato Red”, Vreugdenhil, De Lier, the Netherlands) was grown in 12 compartments (plots; 150 × 100 × 83 cm, L × W × H) in a climate-controlled room at 22°C/19°C air temperature, 16-hour photoperiod, 70% relative humidity, and 800 ppm CO_2_. Seeds were sown into stonewool plug trays (Rockwool Grodan, Roermond, the Netherlands), covered with a layer of vermiculite, kept 24 hours in the dark in the climate chamber, and then exposed to an incident photosynthetic photon flux density (PPFD) of 213 ± 2.2 µmol m^−2^ s^−1^ which was provided by red (R; 600-700 nm) and blue (B; 400-500 nm) LEDs (89% R and 11% B; Philips, GPL PM 210 DRBWFR_R L150 3.1 C4; Phytochrome Photostationary State (PSS) 0.89, based on Sager et al., 1988). Initial incident PPFD was measured at stonewool block height with a quantum sensor (LI-COR, LI-250A Lincoln, NE, USA). On day 19 after sowing, seedlings with two true leaves were transplanted into stonewool blocks (10 × 10 × 7 cm, L × W × H). Side shoots were removed twice a week upon appearance. Plants were pruned to three trusses with nine flowers each and supported with wooden sticks on day 28 after transplanting (DAT). Plants were grown for 100 DAT. Ebb-and-flow irrigation with nutrient solution was supplied twice per week from 0 to 50 DAT and daily from 50 to 100 DAT. The nutrient solution (electrical conductivity 2.1 dS/m, pH 5.5) contained 1.2 mM NH_4_^+^, 7.2 mM K^+^, 4.0 mM Ca^2+^, 1.8 mM Mg^2+^, 12.4 mM NO_3_^−^, 3.3 mM SO_4_^2−^, 1.0 mM PO_4_^2−^, 35 µM Fe^3+^, 8.0 µM Mn^2+^, 5.0 µM Zn^2+^, 20 µM B, 0.5 µM Cu^2+^, and 0.5 µM MoO_4_^2−^.

### 2.2 Treatments

**Figure 1:**
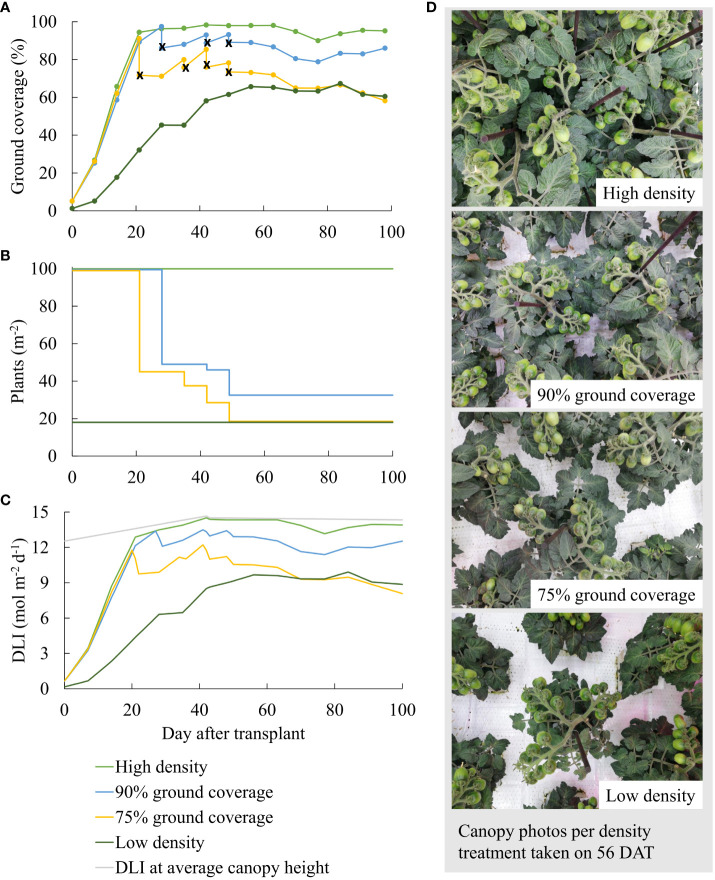
**(A)** Ground coverage, **(B)** resulting planting densities, **(C)** incident (in gray) and intercepted daily light integral (DLI; in colors) per day after transplant, and **(D)** representative canopy photos taken on 56 DAT per density treatment. In **(A)** ground coverage after the spacing of the plants is indicated with an x. In **(C)** the light gray line indicates incident daily light integral (DLI) at average canopy height across all treatments. The density treatments resulted in 100, 54, 41, and 19 plants per m^2^ averaged over the whole growth cycle.

Four treatments, with two constant planting densities (high density and low density) and two dynamic spacing treatments (90% ground coverage [GC] and 75% GC), resulted in 100, 19, 54, and 41 plants/m^2^ averaged over 100 days from transplant to destructive harvest (detailed information on spacing in **Supplementary S1**). High planting density was chosen based on the smallest stonewool block size (10 × 10 × 7 cm) available for growing dwarf tomato. Low planting density was chosen based on information provided by the breeder, who reported a final plant distance of 25 cm for “Plum Tomato Red” plants (Petra Molenaar, pers. comm.), which was implemented in a checkerboard pattern (25-cm interplant distance within rows and 21.6-cm distance between rows). On 0 DAT, the dynamic spacing treatments were arranged identically to the high density. Ground coverage was measured twice per week using the smartphone application “Canopy Cover Free” (based on the “Easy Leaf Area” software, Easlon and Bloom, 2014; **Supplementary S1**). When ground coverage exceeded 90% and 75% for the 90% GC and 75% GC treatments, respectively, plants were manually spaced apart to 90% and 75%, respectively. Spacings, and thus a reduction in ground coverage, took place under 75% GC on 21, 35, 42, and 49 DAT and under 90% GC on 28, 42, and 49 DAT (**Figure 1A**).

### 2.3 Plant morphology

**Figure 2:**
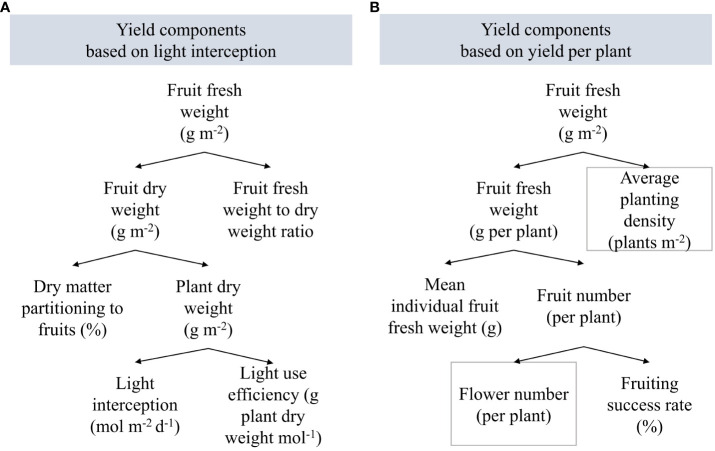
Yield components **(A)** based on light interception, dry matter partitioning, and fresh weight to dry weight ratio, and **(B)** based on fruiting success rate, fruit number and size, and average planting density. Gray boxes indicate fixed parameters.

Truss and flower pruning to three trusses with nine flowers each allowed for a maximum of 27 fruits per plant. Measurements were performed on each of the 12 replicate plants per plot. Flower and green fruit numbers were recorded weekly. Harvest of fully red-ripe fruits took place twice per week from 70 to 100 DAT. Harvested fruits were recorded for each plant. Fruit number and fresh weight were recorded collectively for each truss position per plot. The 100-day and annual harvestable yields (3.65 times 100-day yield) of 1 m^2^ were calculated based on the average planting density and yield per plot (i.e., 12 plants; **Figure 2B**). At each harvest, up to three fruits from the three truss positions per plot were used to determine individual fruit weight and quality parameters. Fruits were dried in a ventilated oven for 48 hours (4 hours at 70°C and 44 hours at 105°C) to determine dry weight. On 99 DAT, the temperatures of a top leaf and the stem base of each plant were measured using an infrared thermometer (Fluke 63 Infrared Thermometer).

On 100 DAT, all remaining ripe and unripe fruits were harvested. Fruits with blossom-end rot were excluded from the harvestable fruit yield and were not considered in the calculation of dry matter partitioning. Green fruits were considered for calculating dry matter partitioning but were not considered as harvestable yield. Before destructively harvesting plants from their plot, dropped leaves were collected from underneath the canopy and included in the leaf dry weight. Plants were dissected into leaves and stems (including trusses), and the existing leaves and leaf scars were counted. Stem diameter was measured using a caliper at the stem base along the smaller side of the cross-section. Stem length from the stem base to the beginning of the top leaf or truss was measured using a measuring tape. Leaf area was determined using an LI-3100 Area Meter (LI-COR, Lincoln, UK) and used to calculate the final LAI. Stonewool blocks, including roots, were discarded. All above-ground plant material was oven-dried for 48 hours (4 hours at 70°C and 44 hours at 105°C).

### 2.4 Yield components and light-use efficiency

Treatment effects on fresh fruit weight were analyzed based on the underlying yield components (**Figure 2**). Total fruit fresh weight refers to green and red-ripe fruit dry weight divided by fruit dry matter content. Total fruit dry weight depends on plant dry weight and assimilate partitioning to fruits. Plant dry weight accumulation is driven by light interception. Plant morphological parameters such as LAI, plant compactness, dry matter allocation, and specific leaf area influence the plants’ efficiency in converting light into biomass. Harvested fruit fresh weight per plant is the product of the red-ripe fruit number per plant and their mean individual fruit fresh weight.

Incident and intercepted LUE were calculated as the ratio of harvested fruit fresh weight (g/m^2^ over 100 days) to the cumulative incident and intercepted PPFD, respectively. Intercepted light was estimated to be the product of the incident daily light integral (DLI; cumulative PPFD per day) and ground coverage, which was measured twice per week and linearly interpolated to obtain daily values. Incident DLI was calculated based on canopy height and PPFD measurements at different canopy heights. Daily canopy height was calculated based on weekly measured canopy height (from 0 to 42 DAT) and individual plant height measurements on 99 DAT and linear interpolation between measurements (**Supplementary S3**). Canopy-incident PPFD increased from 213 ± 2.2 µmol m^−2^ s^−1^ (12.5 mol m^−2^ day^−1^) at the cultivation start to 249 ± 2.8 µmol m^−2^ s^−1^ (14.3 mol m^−2^ day^−1^) at the final average canopy height (indicated as a gray line in **Figure 1C**; **Supplementary S3C**).

### 2.5 Fruit quality

Fruit quality measurements were performed on red-ripe harvested fruits without blossom-end rot that were not located in the first position of the truss. Individual fruit fresh weight, length, diameter, and hardness were measured. Fruit hardness, expressed as the maximum force (N) needed for penetration of the probe (2.5 mm diameter) into the fruit through the tomato skin, was measured using a Zwick machine (Zwick/Roell 2.5kN zwicki RetroLine, Ulm, Germany). Fruits were held in place by a perforated metal cylinder (outer diameter 19.9 mm, inner diameter 8.3 mm, height 23 mm, and hole depth 18 mm). Then, the juice of individual fruits was squeezed into Eppendorf tubes, from which one drop was transferred to the Atago Pocket Brix-Acidity Meter (PAL-BX|ACID Fukaya-Shi, Saitama, Japan) to determine the total soluble solid content (°Brix). The tubes were stored at −80°C. After defrosting, fruit juice was diluted 50-fold with pure water to measure the titratable acid content (% citric acid) using the Atago Pocket Brix-Acidity Meter (Fanwoua et al., 2019; Ji et al., 2019; Ji et al. 2020).

### 2.6 Statistical setup and analysis

The experiment was designed as a randomized complete block design with four treatments and three replications in space, with repetitions in space representing the blocks. Nevertheless, no block effects were found; thus, the data were analyzed using a completely randomized design. Each plot consisted of 12 replicate plants (three rows of four plants), which were surrounded by a row of border plants. The Shapiro–Wilk test was applied to test for the normality of the residuals. Equal variances were assumed due to the low number of replicates. A one-way analysis of variance (ANOVA) was conducted followed by mean separation according to Fisher’s protected least significant difference (LSD) test (p = 0.05). In one occasion (intercepted DLI per plant in **Table 1**), the normality of residuals was rejected by the Shapiro–Wilk test; the QQ plot was examined visually, and the data were deemed adequate to perform a subsequent ANOVA.

**Table 1:** Plant morphological parameters, canopy-incident daily light integral (DLI), and intercepted DLI per plant.

	High density	90% GC	75% GC	Low density	p-Value of F-statistic
Canopy height averaged over 100 days (cm)	20.6 a	19.5 b	18.3 c	18.4 c	0.00^***^
Incident light intensity averaged over 100 days (mol m^−2^ day^−1^)	14.4 a	14.2 b	14.1 c	14.1 c	0.00^***^
Intercepted light intensity averaged over 100 days (mol day^−1^ per plant)	0.12 c	0.20 b	0.22 b	0.38 a	0.00^***^
Internode length (cm)	2.4 a	2.1 ab	2.0 b	1.9 b	0.02^*^
Basal stem diameter (mm)	6.1 c	8.5 b	9.0 a	9.0 a	0.00^***^
Slenderness (%; cm/cm)	0.4 a	0.3 b	0.2 c	0.2 c	0.00^***^
Specific stem length (cm/g)	11.3 a	5.2 b	4.1 bc	3.7 c	0.00^***^

Letters indicating significant differences [least significant difference (LSD) test] between treatments and the p-value of the F-statistic are provided (^*^p < 0.05 and ^***^p < 0.001). Data are the means over three blocks (n = 3), each with a canopy consisting of 12 replicate plants. The density treatments resulted in 100, 54, 41, and 19 plants per m^2^ on average.

GC, ground coverage.

## 3 Results

### 3.1 High planting density increased light interception, especially during early cultivation

Higher planting density resulted in higher ground coverage and hence higher light interception, particularly early during cultivation (**Figure 1**). Incident PPFD at the canopy top slightly increased when the canopy grew in height. Canopy height was slightly larger at high density (**Table 1**).

Ground coverage was used as a proxy for the fraction of the intercepted PPFD. The initial increase in ground coverage over time was due to the rapid leaf expansion of young plants (0–20 DAT in **Figure 1A**). From 21 DAT, manual spacing primarily determined ground coverage differences between the density treatments. During fruiting (75–100 DAT), numerous fruits were located on top of the canopy and were (falsely) detected as leaf areas while they were green and as non-leaf areas while they were red.

### 3.2 Higher light interception resulted in more plant and fruit biomass

**Figure 3:**
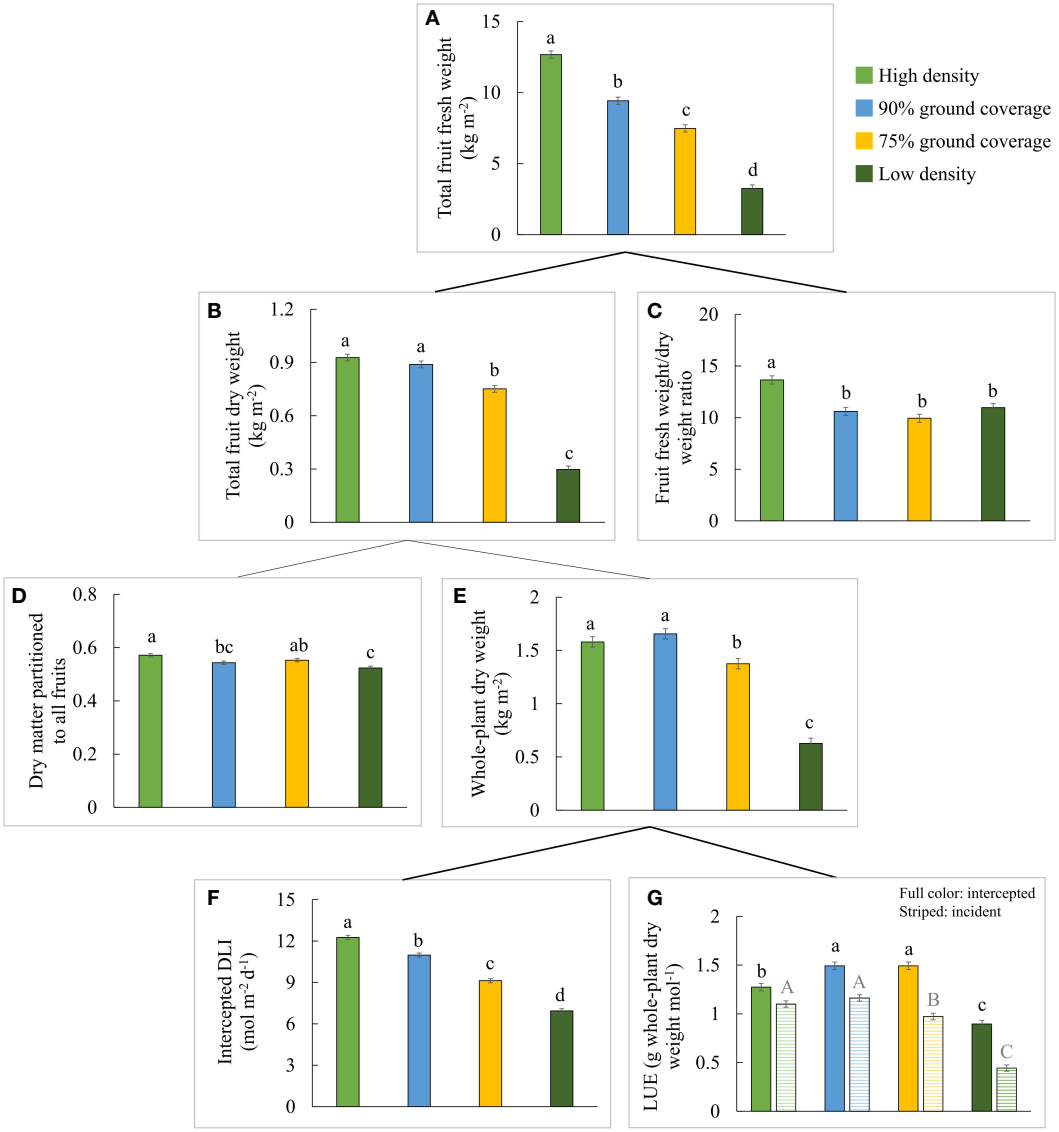
Effects of planting density on yield components constituting the total fruit fresh weight of all (red-ripe and green) fruits from a 1-m^2^ cultivation area over 100 days: **(A)** total fruit fresh weight, **(B)** total fruit dry weight, **(C)** fruit fresh weight to dry weight ratio, **(D)** dry matter partitioned to fruits, **(E)** cumulative whole-plant dry weight, **(F)** intercepted daily light integral (DLI), and **(G)** intercepted (full color) and incident (striped) whole-plant light use efficiency. The letters indicate significant differences [least significant difference (LSD) test, p = 0.05]. Data are the means over three blocks (n = 3), each with a canopy consisting of 12 replicate plants, surrounded by border plants. Error bars indicate standard errors of means. The density treatments resulted in 100, 54, 41, and 19 plants per m^2^ on average.

Differences in light interception (**Figures 1C**, **3F**) were primarily caused by differences in ground coverage, which decreased by 43% at low density compared to high density. Differences in incident PPFD were less of a consequence of differences in plant height, which varied by a maximum of 11% (75% GC compared to high density in **Table 1**). In addition to the resulting highest light interception (**Figure 3F**), a constant high planting density produced the highest total fruit fresh weight per m^2^ (**Figure 3A**), although the total fruit dry weight per m^2^ was not significantly different from 90% GC (**Figure 3B**). Whole-plant dry weight per m^2^ was the highest at high density and 90% GC (**Figure 3E**). The efficiency of a plant to convert light that was intercepted at the top leaves to plant dry weight was highest at 90% and 75% GC, lower at high density, and lowest at low density (**Figure 3G**). Whole-plant incident LUE was higher at higher planting densities (**Figure 3G**).

**Figure 4:**
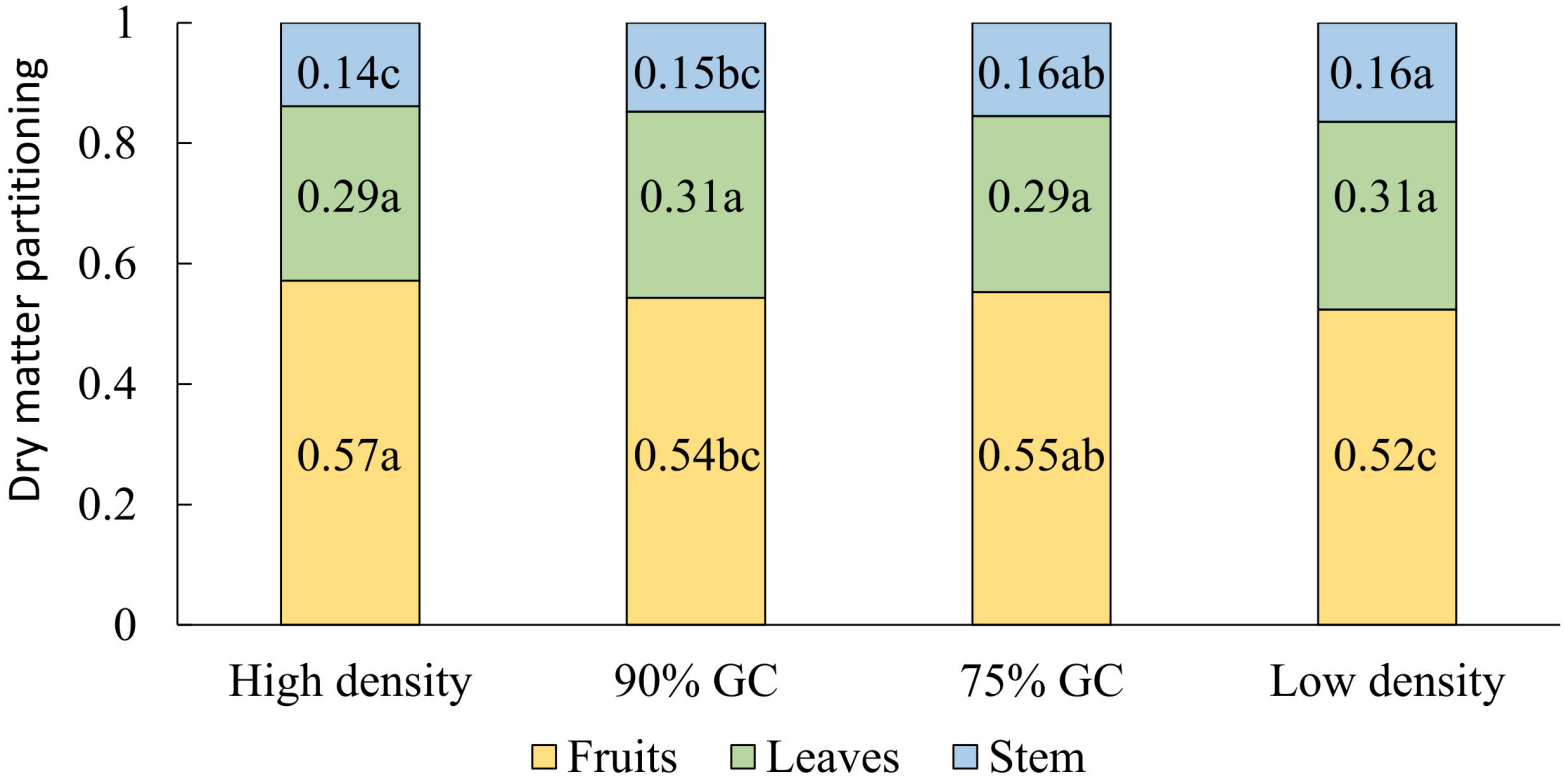
Effects of planting density on the fraction of dry matter partitioned among all (red-ripe and green) fruits, stem plus trusses, and leaves. Means followed by different letters indicate significant differences [least significant difference (LSD) test, p = 0.05]. Data are the means over three blocks (n = 3), each with a canopy consisting of 12 replicate plants. The density treatments resulted in 100, 54, 41, and 19 plants per m^2^ on average. GC, ground coverage.

A slightly higher partitioning of dry matter to fruits was observed at high density compared to low density (**Figure 3D**). Dry matter partitioned to the leaves did not differ significantly between the density treatments, but dry matter partitioned to the stem increased from high to low density (**Figure 4**). Absolute stem dry weight and absolute leaf dry weight per plant on day 100 were more than doubled under all other densities compared to high densities.

### 3.3 High planting density resulted in the highest fruit yield per area despite fruit yield reductions per plant

**Figure 5:**
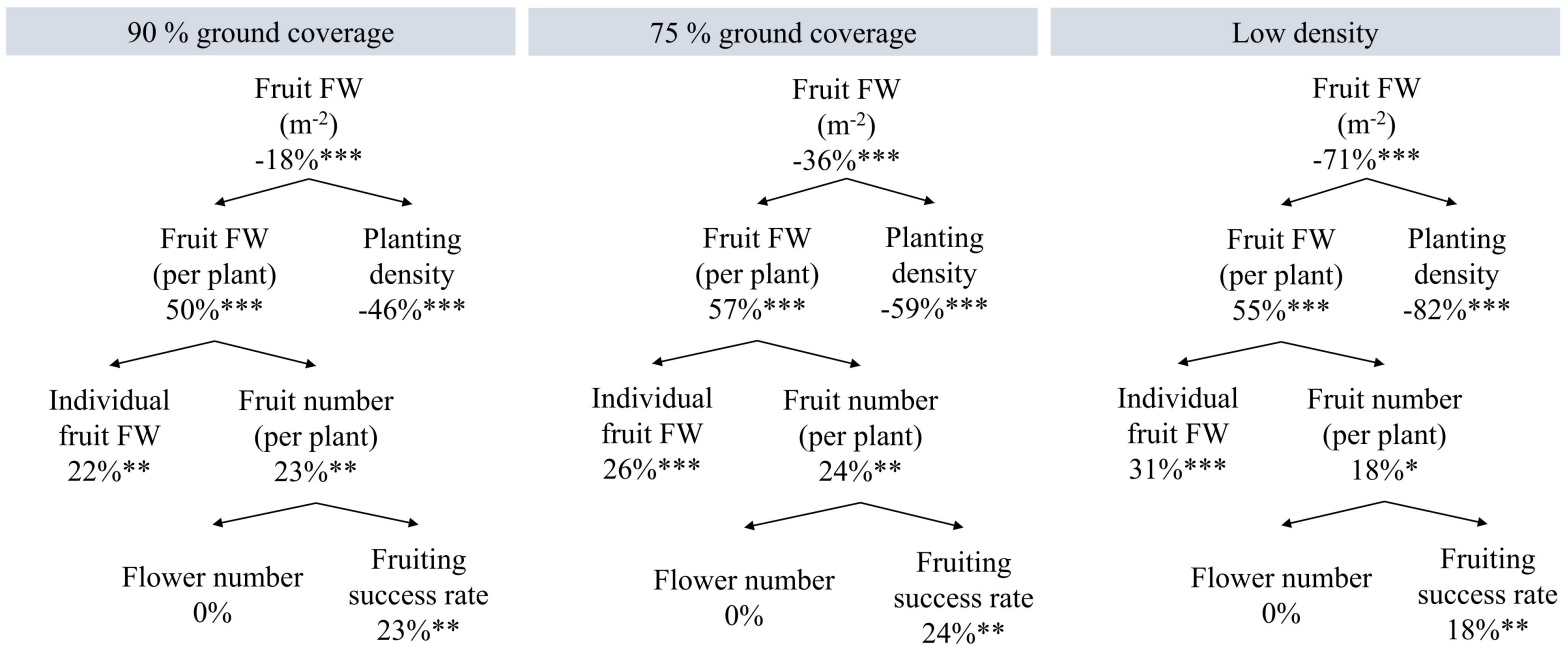
Effects of planting density on yield components constituting fruit fresh weight (FW) of red-ripe harvested fruits from a 1-m^2^ cultivation area over 100 days. Percentages are the increments compared to high density. High density resulted in 11.1 kg fruit FW m^−2^, 111 g fruit FW per plant; 100 plants/m^2^ average planting density; 19.4 harvested fruits per plant; 5.7 g average FW per fruit; 27 flowers per plant (plants had been pruned to 27 flowers); and a 72% flower fruiting success rate. *p < 0.05, **p < 0.01, and ***p < 0.001. Data are the means over three blocks (n = 3), each with a canopy consisting of 12 replicate plants.

High density resulted in 11.1 kg/m^2^ fruit weight of red-ripe harvested fruits over 100 days. Thus, 40.5 kg/m^2^ annual harvestable fruit yield can be obtained under continuous high-density cultivation. Compared to high density, the dynamic spacing and low-density treatments resulted in 18%, 36%, and 71% reductions in red-ripe fruit fresh weight per m^2^ (**Figure 5**; **Supplementary S4**). At the plant level, high density resulted in reduced productivity: it had the lowest fruit fresh weight per plant, individual fruit fresh weight, fruit number per plant, and also the lowest fraction of flowers that turned into harvested ripe fruits (fruiting success rate; i.e., most flower abortion and non-ripened fruits on 100 DAT). High density yielded on average 2.83 unripe fruits per plant on day 100, more than 90% GC (0.94 unripe fruits), 75% GC (0.94 unripe fruits), and low density (0.56 unripe fruits). No significant density-driven temperature differences were observed: on 98 DAT, neither the temperatures of leaves at the top of the canopy nor those of stems close to its base were statistically different between the treatments. There was no noticeable difference between treatments regarding the timing of flowering and fruit ripening (**Supplementary S5**).

### 3.4 Plants grown at higher planting densities converted light more efficiently into fruit yield

**Figure 6:**
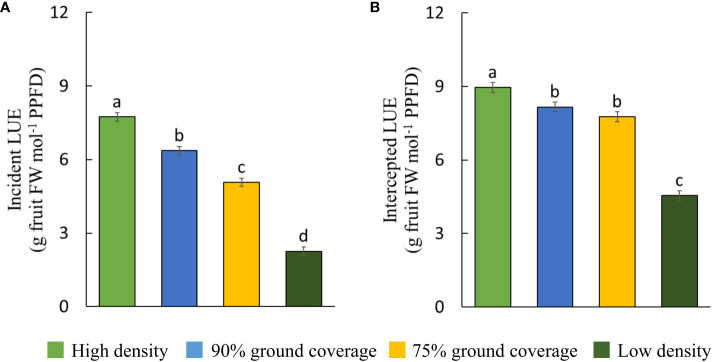
Light use efficiency (LUE) of **(A)** incident and **(B)** intercepted photosynthetic photon flux density per density treatment. Fruit fresh weight refers to harvested red-ripe fruits over 100 days. The letters indicate significant differences [least significant difference (LSD) test, p = 0.05]. Data are the means over three blocks (n = 3) each with a canopy consisting of 12 replicate plants. Error bars indicate standard errors of means. The density treatments resulted in 100, 54, 41, and 19 plants per m^2^ on average.

**Figure 7:**
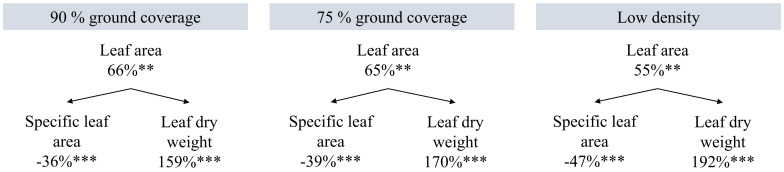
Effects of planting density on leaf area, specific leaf area, and leaf dry weight per plant on day 100. Percentages are the increments compared to high density. High density resulted in 733 cm^2^ leaf area per plant, 203 cm^2^/g specific leaf area, and 3.6 g leaf dry weight per plant. **p < 0.01, and ***p < 0.001. Data are the means over three blocks (n = 3) each with a canopy consisting of 12 replicate plants.

Higher planting density resulted in a higher efficiency of plants in converting incident and intercepted PPFD into red-ripe fruits (**Figure 6**). Intercepted DLI per plant was more than tripled (+206%) at low density compared to high density (**Table 1**), while fresh fruit weight per plant was 55% higher (**Figure 5**). Internode elongation with increasing density showed that plants were less compact at high density (**Table 1**). Stem diameter was reduced at high density (**Table 1**). Thus, plant slenderness (height of plants over diameter of stems) and specific stem length (stem length divided by stem mass) were higher at high density (**Table 1**). At low density, leaf area per plant was 55% and leaf dry weight 192% higher than at high density, resulting in reductions in specific leaf area (**Figure 7**). Each plant initiated on average 10.8 leaves, with no significant differences between treatments. High-density plants had dropped on average 4.9 of their lowest leaves until day 100, thus resulting in a higher observed dry matter partitioning to fruits compared to leaves at the final harvest. Leaf dropping occurred less in the other densities (maximum 0.75 leaves per plant under low density). LAI on day 100 (excl. senescent leaves) was highest at high density (6.7), lower at 90% GC (3.9), and lowest at 75% GC and low density (2.1 and 2.2, respectively).

### 3.5 Dynamic spacing mitigated fruit quality-reducing effects of high planting density

Fruit quality was reduced at high density (**Table 2**). No or marginal fruit quality differences were observed between 90% GC, 75% GC, and low density. Individually measured fresh fruit weight and fruit size were reduced at high density. Individual fruit weight measurements were consistent with the results of the calculated individual fresh fruit weight (total fresh fruit weight divided by fruit number; **Figure 5**). At high density, fruit hardness, total soluble solid content, and citric acid content were the lowest. The ratio between soluble solids and acidity was not significantly different between treatments. Blossom-end rot in red-ripe fruits hardly occurred; however, more fruits were affected at low density (2.5%) compared to all other densities (0.3%–0.5%). The variability of fruit length and diameter within each plot did not differ significantly between planting densities, but total soluble solid content was less uniform at constant high density compared to all lower densities (**Table 2**).

**Table 2:** Fruit quality parameters of red-ripe fruits based on individual fruit measurements per density treatment between 70 and 100 DAT (total fruit n = 835, with high-density n = 181, 90% GC n = 218, 75% GC n = 226, and low-density n = 210).

	High density	90% GC	75% GC	Low density	p-Value of F-statistic
Fruit weight (g per fruit)	6.0 b *CV 0.15 a*	7.3 a *CV 0.11 b*	7.4 a *CV 0.13 ab*	7.6 a *CV 0.13 ab*	0.00^***^ *0.11 ^ns^*
Fruit length (mm)	22.7 b *CV 0.06 a*	24.8 a *CV 0.05 a*	25.0 a *CV 0.05 a*	25.0 a *CV 0.05 a*	0.00^***^ *0.50 ^ns^*
Fruit diameter (mm)	21.1 b *CV 0.07 a*	22.4 a *CV 0.06 a*	22.3 a *CV 0.07 a*	22.8 a *CV 0.07 a*	0.00^***^ *0.40 ^ns^*
Fruit hardness (N_max_)	5.5 c *CV 0.21 b*	7.2 ab *CV 0.23 b*	7.4 a *CV 0.23 ab*	6.7 b *CV 0.26 a*	0.00^***^ *0.07 ^ns^*
Total soluble solids (°Brix)	6.6 c *CV 0.11 a*	8.9 b *CV 0.06 b*	9.4 a *CV 0.05 b*	9.4 a *CV 0.04 b*	0.00^***^ 0.00^***^
Titratable acids (% citric acid)	0.8 b *CV 0.25 a*	1.0 a *CV 0.27 a*	1.0 a *CV 0.26 a*	1.0 a *CV 0.24 a*	0.00^***^ *0.81 ^ns^*
Ratio of total soluble solids to titratable acid	9.4 a *CV 0.33 a*	9.6 a *CV 0.34 a*	9.8 a *CV 0.36 a*	9.9 a *CV 0.25 a*	0.58 ^ns^ *0.55 ^ns^*
Blossom end rot (% of all red-ripe harvested fruits)	0.5 b	0.3 b	0.5 b	2.5 a	0.07 ^ns^

Letters indicating significant differences [least significant difference (LSD) test] between treatments and the p-value of the F-statistic are provided (^***^p < 0.001). Data are the means over three blocks (n = 3) each with a canopy consisting of 12 replicate plants. The coefficient of variation (CV) within a plot is shown in italics. The density treatments resulted in 100, 54, 41, and 19 plants per m^2^ on average.

GC, ground coverage; ns, non-significant difference.

## 4 Discussion

### 4.1 Maintaining high ground coverage based on canopy photos improves space and light use

Dynamic spacing can be used to maintain high ground coverage to allow for efficient use of incident light while avoiding competition-induced assimilate shortages at the plant level. Much light is lost when plants are young and do not intercept all available light. Light is also lost after seedlings are transplanted to pots or substrate blocks. Providing far-red light (700–750 nm) can accelerate early leaf expansion, canopy closure, and subsequent light interception (Jin et al., 2021; Carotti et al., 2023) until dynamic spacing is adequate to control ground coverage.

In our experiment, growing transplanted seedlings at the maximum possible density resulted in faster canopy closure and higher daily light interception than under low density (**Figure 1C**). The steep increase in ground coverage under 100 plants/m^2^ during early cultivation indicates that dynamic spacing is advantageous over growing plants constantly at the spacing treatment’s average planting density (e.g., constant 54 plants/m^2^ instead of step-wise decreasing density from 100 to 32.5 plants/m^2^ at 90% GC; **Supplementary Table S1C**). Accelerating canopy closure during early cultivation by growing at high planting density allows for higher intercepted DLI (**Figure 1**) and whole-plant LUE (**Figure 3G**), which result in more biomass and fruit yield per cultivation area (**Figures 3E, A**). Nevertheless, planting density must be reduced before plants develop yield-reducing responses to high densities, such as flower abortion (Papadopoulos and Ormrod, 1991). By maintaining 90% ground coverage, a percentage close to 100%, we aimed for a high harvestable fruit yield without reducing quality.

Ground coverage was measured based on bird’s-eye view photos of the canopy, which is easily applicable, fast, and non-destructive. Yet, actual light interception occurs at multiple leaf layers, not only at the top leaves. We decided against dynamic spacing based on LAI since LAI determination requires regular destructive measurements and lacks practicability for implementation in commercial vertical farms. Utilizing the app “Canopy Cover Free”, ground coverage was determined based on the ratio of green (500–600 nm) to red (600–700 nm) in the photo (Easlon and Bloom, 2014).

### 4.2 Individual plants experience assimilate shortage at very high planting densities

Light interception depends on the percentage of cultivation area covered by plants. In line with Postma et al. (2021), we showed that planting density affects plant dry weight per cultivation area (**Figure 3E**) and light interception (**Figure 3F**). Under constant high planting density, the final whole-plant dry weight per area was not significantly higher than at 90% GC, which indicates a lower assimilate availability per plant. Further, the dropping of lower, shaded leaves at high density caused an underestimation of the total dry weight produced per plant over 100 days. Dropped leaf biomass was collected from underneath the dense canopy prior to the destructive harvest, but in the meantime, leaf dry weight was lost through decomposition. Lost leaf biomass resulted in an underestimation of dry matter partitioning to leaves and an overestimation of dry matter partitioning to fruits and stems (**Figures 3D**, **4**). Under constant high density, dry matter partitioned to fruits was higher compared to lower densities, but the fruit number per plant was lower. Thus, we propose that the sink strength of individual fruits may have increased under constant high density. Distal fruits on a truss were reported to have a lower fruit sink strength than fruits located higher on the truss (Bertin, 1995). At high density, mostly distal flowers were aborted. Consequently, individual fruit sink strength at high densities may have been higher than under lower densities.

### 4.3 Flower abortion and reduced assimilate availability limit fruit yield per plant at high planting density

Higher planting density resulted in higher plant dry weight, fruit dry weight, and fruit fresh weight per cultivation area (**Figures 3E, B**, **5**). Similarly, harvestable greenhouse tomato yield per area was reported to increase under constantly higher (Heuvelink, 1995) or dynamically increased stem densities (Cockshull and Ho, 1995). Postma et al. (2021) found that total and generative dry weight per area increases with planting density, but only until a density threshold is exceeded and plant-level resource deficiencies inhibit growth. In our experiment, whole-plant dry weight per cultivation area (**Figure 3E**) and productivity per plant (**Figure 5**) showed those density-driven limitations under constant high density. For instance, the lowest flowers per plant (max. 27) developed into red-ripe fruits compared to all other densities due to higher flower abortion, higher breaking-off of green fruits, and possibly slower ripening (**Figure 5**; **Supplementary Figure S5B**). The latter indicates that plant development was slowed down by a few days at constant high density; thus, marginally more harvestable fruit yield could be obtained with a longer cultivation cycle for the remaining green fruits to turn fully red, but at the expense of LUE.

Interestingly, we did not find a significant vertical temperature gradient within the canopies. Still, temperature gradients within dense canopies can be expected, especially since controlled environments are generally characterized by lower wind speeds compared to open-field production (Poorter et al., 2016). Postma et al. (2021) mentioned a possible (temperature-driven) 2% increase in days until flowering for a wide range of species when planting density is doubled. Source-sink ratios rarely affect flowering rate (de Koning, 1994). However, possibly, fewer assimilates were available to distal fruits on the truss (Bertin, 1995)—like the ones that remained green until day 100 under the high-density treatment (**Figure 5**).

Assimilate partitioning to the fruits and, consequently, the harvest index (HI; fruit dry weight over total above-ground plant dry weight) in tomatoes is impacted by crop maintenance. For instance, side-shoot removal and pruning affect plant morphology and biomass allocation in the plant. Langenfeld and Bugbee (2023) reported HI (based on fresh weight) ranges of <8% to 46% for eight dwarf tomato cultivars (note that these fractions would have been lower when expressed on a dry mass basis, as the dry matter content of tomato fruits is lower than that of leaves and stems). Those eight cultivars received no side-shoot removal (Noah Langenfeld, pers. comm.), which certainly resulted in higher assimilate partitioning to vegetative plant organs and reduced HI. In our experiment, dry matter partitioning to fruits was above 50% for all densities.

### 4.4 Plant acclimation to high planting densities increases light use efficiency

Generally, it is expected that incident LUE increases with higher planting densities (**Figure 6A**; Jin et al., 2023). Postma et al. (2021) found that yield reductions per cultivation area when doubling planting densities are smaller than reductions in resource use (e.g., space and light); thus, resource use efficiency increases. We observed marginal increases in incident PPFD due to a slight increase in canopy height with increasing planting density and because PPFD was higher closer to the lamps (**Table 1**). Within a canopy, most incident light is usually intercepted at an LAI of three to four in a wide range of crops, with hardly any gain at higher LAIs (Heuvelink et al., 2004; Postma et al., 2021). At the final harvest, high density resulted in an excessively high LAI despite the prior dropping of leaves, 90% GC in a desirable LAI, and 75% GC and low density in low LAIs.

Maximum reported incident LUE ranges from 1.26 to 1.81 g dry weight per mol photons for canopies that intercept 90%–95% of incident light (e.g., Loomis and Williams, 1963; Zhu et al., 2010; in Jin et al., 2023). Whole-plant incident LUEs for dwarf tomato grown over 100 days under dynamic and constantly high planting densities (1.16 g/mol under 90% GC and 1.10 g/mol at high density) are close to the maximum reported LUE. This may be possible due to the achievement of high instantaneous incident LUEs during early cultivation, which are usually—at constant and lower planting densities—low at transplant and increase with leaf expansion until final harvest (Jin et al., 2023).

In this study, the morphological responses to high planting densities differ from the mentioned references, e.g., from the reviews on shade avoidance (Franklin, 2008) and planting density (Postma et al., 2021), in that there was no solar light—importantly, no far-red light— present in the applied red-blue light spectrum. Also, root zone competition in the stonewool blocks was assumed to be absent and was not assessed. Typical shade avoidance responses of plants grown in soil and under solar light are adaptations to complex interactions of reduced light intensity, reduced red-to-far-red ratio within shaded canopies (e.g., Franklin, 2008), and reduced nutrient availability at the plant level. Here, high-density responses were attributed mainly to differences in PPFD.

### 4.5 Assimilate shortage reduces fruit quality at high planting density

High planting density results in lower individual fruit weight and size, more fruit size variability, and therefore reduced marketability (Cockshull and Ho, 1995; Heuvelink, 1995). We observed a reduction in fruit size, but—contrary to Cockshull and Ho (1995)—no increase in fruit size variability under constant high density (**Table 2**), which was likely due to truss pruning. A dynamic high planting density (maintaining 90% ground coverage) did not significantly decrease fruit size.

Under constant high density, the total soluble solid content (i.e., sweetness) and citric acid content of red-ripe harvested fruits were reduced (**Table 2**), and fruit dry matter content increased (**Figure 3C**). Nevertheless, the sweetness was shown to decline with decreasing fruit size due to a positive correlation between fruit size and the source–sink ratio (Li et al., 2015). The seed company Vreugdenhil aims for 7°Brix (Jan van Heijst, pers. comm.) in this cultivar, which was not achieved at high density, presumably due to assimilate shortage at the plant level. Red-ripe harvested cherry tomatoes were reported to range from 4.5 to 6.9°Brix and 0.1% to 1.4% citric acid content (Borba et al., 2021; del Carmen Damas-Job et al., 2023; Pattanapo et al., 2023), resulting in a sweetness-to-acidity ratio of 10 to 100, a ratio that is slightly higher than we obtained (9.4 to 9.9; **Table 2**). Malundo et al. (1995) found that perceived tomato flavor benefits from higher sweetness if citric acid content is ≥0.8%. To enhance sweetness, far-red light can be applied to increase dry matter partitioning in fruits and subsequent sugar accumulation in fruits (Fanwoua et al., 2019; Ji et al., 2019; Ji et al., 2020).

Blossom-end rot was observed rarely, but most often at low density. The absence of a closed canopy resulted in higher light interception, likely leading to higher transpiration rates per plant and higher growth rates, thus causing deficiencies in calcium (Hagassou et al., 2019).

Fruit hardness, besides flesh firmness, constitutes fruit texture. Hardness positively influences shelf-life (Beckles, 2012) and perceived visual tomato quality (i.e., surface smoothness; Batu, 2004). The hardness of red-ripe cherry tomatoes at harvest is ca. 2 N (del Carmen Damas-Job et al., 2023; cf., table grapes range from 6 to 11.7 N; Deng et al., 2005; Rolle et al., 2011; Balic et al., 2022). Thus, the observed hardness under all planting densities (**Table 2**) is within the reported ranges of comparable fruit crops.

### 4.6 Implementation and future research

The present experiment is proof of the concept that dynamic spacing results in high harvestable fruit yields while mitigating the fruit quality-reducing effects of high planting densities. For implementation, this concept can be tested on a larger scale, extended to different light spectra and other varieties and crops, and in combination with different pruning techniques. Also, dynamic light management, dynamic climate control, and their interactions should be explored to improve resource use efficiency (i.e., energy and space use efficiency) and thus the environmental and economic performance of highly controlled crop production systems. Finally, the economic cost–benefit ratio of spacing must be determined, where benefits are related to yield, and costs are related to increased labor and/or automation. The cost-effectiveness of manual versus automated spacing depends, among others, on the scale of production and the costs of labor and equipment. Ideally, the capacity for automated spacing is considered during the design phase of a new production unit.

## 5 Conclusions

Our results demonstrate that high ground coverage and thus high light interception throughout cultivation are key to maximizing both yield per area and LUE: plants grown under constant high planting density utilized light most efficiently for fruit yield formation (11.1 kg/m^2^ with an LUE of 7.7 g yield mol^−1^ photons incident on the canopy) due to a rapid canopy closure and then consistently high ground coverage of ~96%. Nevertheless, fruit quality was reduced under constant high density. As hypothesized, constant low planting density resulted in the lowest light interception, which resulted in the lowest yield per cultivation area (3.2 kg/m^2^) and the lowest LUE (2.3 g yield mol^−1^). Dynamic spacing—i.e., growing plants initially at a high planting density but then spacing them apart to maintain constant ground coverages of 75% and 90%—resulted in the same fruit quality, but more than double the yield compared to low density. Thus, dynamic spacing mitigates density-induced trade-offs between fruit yield and quality in dwarf tomato.

## Data availability statement

The original contributions presented in the study are included in the article/**Supplementary Material**. Further inquiries can be directed to the corresponding author.

## Author contributions

MK: Conceptualization, Data curation, Formal analysis, Investigation, Visualization, Writing – original draft, Writing – review & editing. LM: Conceptualization, Funding acquisition, Methodology, Writing – review & editing. EH: Conceptualization, Funding acquisition, Methodology, Writing – review & editing.
