# Supplementary material for: Dynamic plant spacing in tomato results in high yields while mitigating the reduction in fruit quality associated with high planting densities
Source: Front Plant Sci. 2024 Apr 18;15:1386950. doi: 10.3389/fpls.2024.1386950 (PMC11063277; doi:10.3389/fpls.2024.1386950)
Supplement: Supplementary file 1 [file DataSheet_1.pdf]

Supplementary Material

1 Supplementary S1 – Additional information on cultivation area dimensions, dynamic plant spacing, and average planting density

The same spacing was applied to all three plots of the respective spacing treatment at the expense of individual plot' coverage accuracy. High density was grown with 25 cm interplant distance in a checkerboard pattern (21.6 cm distance between rows, 25 cm interplant distance within each row). 90% GC, 75% GC and low density were grown in a regular rectangular pattern with an initial 10 cm interplant distance (between rows and within each row). The final shift on 49 DAT under 90% GC resulted in the identical off-set checkerboard pattern as under high density. Table S1A shows the measured values of ground coverage over time as shown in Figure 1A. Table S1B shows the cultivation area used by a single plant. Table S1C shows the number of plants per m^2 over time and its average over 100 days. After 49 DAT, ground coverage was no re-adjusted (i.e., plants spaced closer together again) although percentages lower than 75% or 90%, respectively, were detected. This reduction in ground coverage was due to the increasing number of ripening fruits located higher than the top leaf layer.

Table S1A. Values of ground coverage (GC; %) over time as shown in Figure 1A. Data is the average of the three plots per treatment. When manual spacing was applied, ground coverage before and after the shift is given. The density treatments resulted in 100, 54, 41 and 19 plants per m^2 on average.

Day after transplant	High density	90% GC	75% GC	Low density
0	5.2	5.1	5.2	1.3
7	26.8	25.3	26.1	5.2
14	65.8	58.6	62.1	17.7
21 before	94.4	89.3	91.1	32.2
21 after	94.4	89.3	71.7	32.2
28 before	96.3	97.5	71.1	45.3
28 after	96.3	86.1	71.1	45.3
35 before	96.5	88.0	80.0	45.3
35 after	96.5	88.0	75.8	45.3
42 before	98.3	93.0	85.4	58.2
42 after	98.3	88.7	75.9	58.2
49 before	97.9	93.2	78.2	61.6
49 after	97.9	89.1	73.4	61.6
56	98.0	89.0	73.2	65.7
63	98.0	86.7	71.9	65.3
70	94.8	80.5	65.0	63.4
77	90.0	78.8	64.9	63.3
84	93.6	83.3	66.5	67.3
91	95.5	83.0	62.3	61.5
98	95.2	86.0	58.2	60.5

Table S1B: Cultivation area (cm^2) covered by one plant per day after transplant. GC = ground coverage (%).

Day after transplant	High density	90% GC	75% GC	Low density
0-21	100	100	100	541.3
21-28	100	100	222	541.3
28-35	100	204	222	541.3
35-42	100	204	266	541.3
42-49	100	217.6	348.9	541.3
49-100	100	308	541.3	541.3
**sum**	**10000**	**23000**	**37300**	**54130**

Table S1C: Planting density (number of plants per m^2) of dwarf tomato plants under constantly high planting density, 90% ground coverage (GC), 75% GC, and constantly low planting density cultivation area during 100 cultivation days.

Day after transplant	High density	90% GC	75% GC	Low density
0-21	100	100	100	18.5
21-28	100	100	45.1	18.5
28-35	100	49	45.1	18.5
35-42	100	49	37.6	18.5
42-49	100	46	28.7	18.5
49-100	100	32.5	18.5	18.5
**mean**	**100**	**54.4**	**41.1**	**18.5**

2 Supplementary S2 – Additional information on ground coverage determination

Ground coverage was measured in-situ with the freely available smartphone application “Canopy Cover Free” which determined the ratio of green area to cultivation area based on the “Easy Leaf Area” software (Eason 2014). The application made use of the smartphone camera (Huawei Mate 10 Lite) and allowed for determining a representation of the ratio of green to non-green pixels of the taken photo after modifying the photo’s color ratios. No green to red ratio could be detected under the cultivation light recipe which consisted of red and blue only. A measurement light recipe was developed with high percentage of green (47% green, 29% blue, 24% red; 3% additional far-red). Settings of the “Canopy Cover Free” application (i.e., green minimum, green to blue ratio, and green to red ration) were kept as constant as possible, yet were moderately adjusted based on subjective assessment per plot to adequately represent reality. Green minimum was always set to 0. Green to blue ratio ranged from 0.8-1, and green to red ratio from 0.92-1.06. Notably, “Canopy Cover Free” also detects green fruits that are located higher than the top leaves as green “leaf” area. Since the green fruits that were “falsely” detected as leaf area were located mostly on top of existing leaves, we assume that detecting green fruits as “covered area” does not impede our results.

When relying on camera vision to detect ground coverage, lens distortion must be considered. Either a camera with minimal distortion must be chosen or a distortion correction must be applied. Camera distortion was assumed to be minor due to the smartphone’s wide-angle camera and was not corrected for in this experiment. The camera position in the center of each plot at 57cm height (50 cm above stonewool block height) detected the cultivation area of experimental plants grown under high density and 90% GC in a representative manner. For low density, two camera positions (right and left of the central position) at 57 cm height resulted in non-overlapping representative canopy photos of the respective side of the plot. For the spacing treatment 75% GC, measurements were taken initially from the central position and after respacing on DAT 49, which resulted in the same cultivation area used per plant as low density, from the right and left camera position. On each measurement day, ten photos per camera position were analyzed and the average per plot calculated. The spacing treatments were spaced apart based on plot averages.

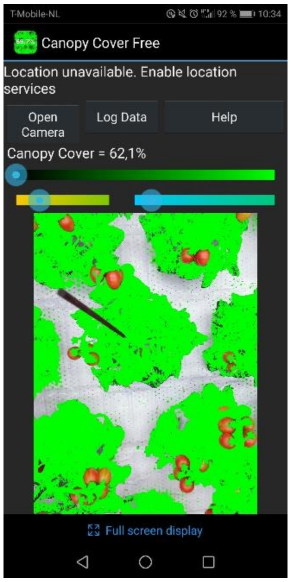

Figure S2A: Screenshot from Canopy Cover Free app showing a canopy cover percentage detection, color correction sliders, and the resulting in a visual representation of green area.

3 Supplementary S3 – Canopy height and daily incident light intensity calculations

Canopy height was measured weekly between 0-42 DAT after which it remained largely unchanged. On 99 DAT, plant height of all experimental plants was measured and averaged per plot to obtain the canopy height. Daily canopy height was calculated using linear regression per treatment averages between 0-42 DAT (Figure S3A) and also between 42-100 DAT (Figure S3B).

Cumulative incident light intensity over the 100 DAT was calculated based on daily canopy height per plot according to the equations shown in Figure S3A and S3B, and based on the assumption that incident light intensity changes linearly with canopy height according to the equation in Figure S3C:

(1) $I_{inc}(t) = 1.64 * h(DAT) + 212.77$

whereas I_{inc} is incident light in µmol m^{-2} s^{-1} at each plot's average canopy height, and h(t) is the average canopy height at a certain DAT in cm.

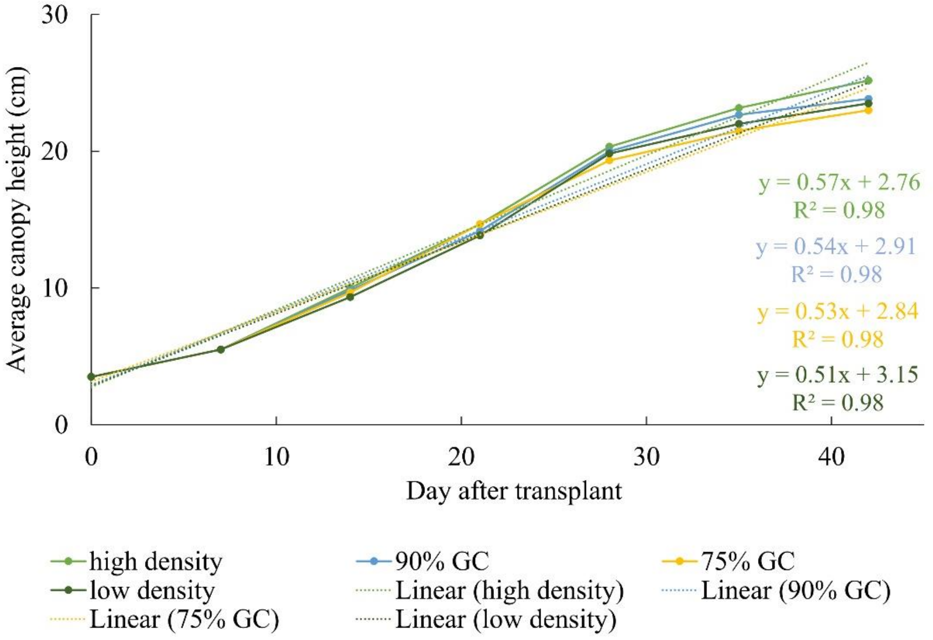

Figure S3A: Average canopy height per treatment from 0 to 42 DAT measured from rockwool block height. GC = ground coverage.

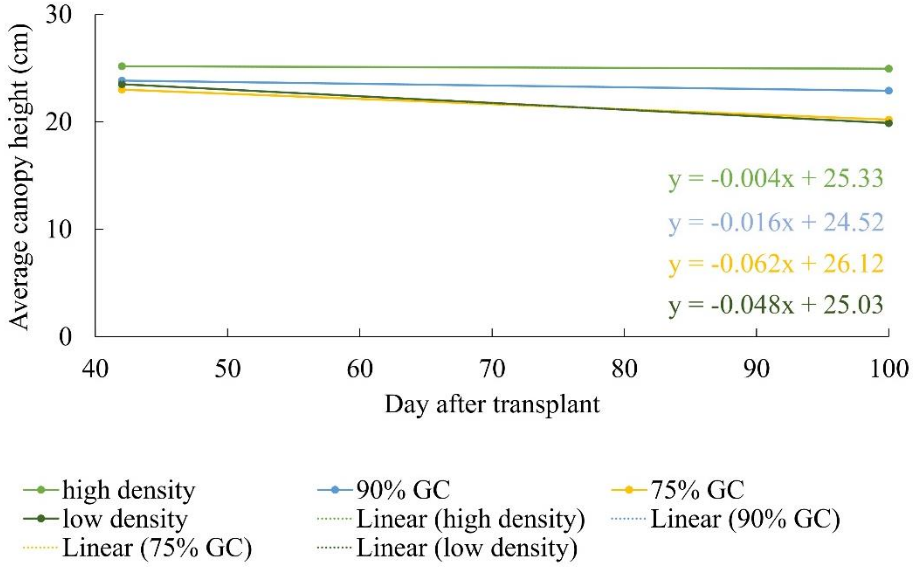

Figure S3B: Average canopy height per treatment from 42 to 100 DAT measured from rockwool block height. GC = ground coverage.

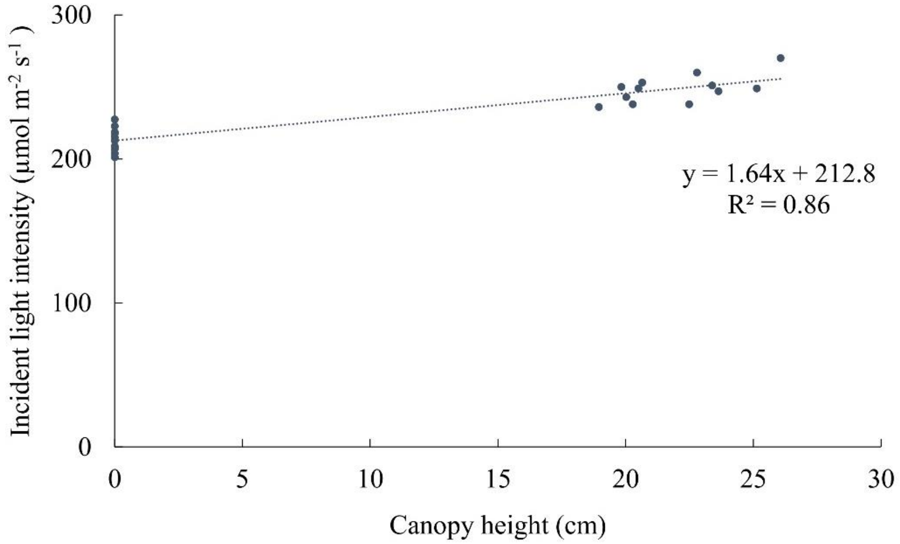

Figure S3C: Incident light intensity at rockwool block height (0 cm) and the final mean canopy height per plot (n = 12; overall final mean height = 21.98 cm).

4 Supplementary S4 – Additional figure supporting yield component analysis on harvestable fruit yield

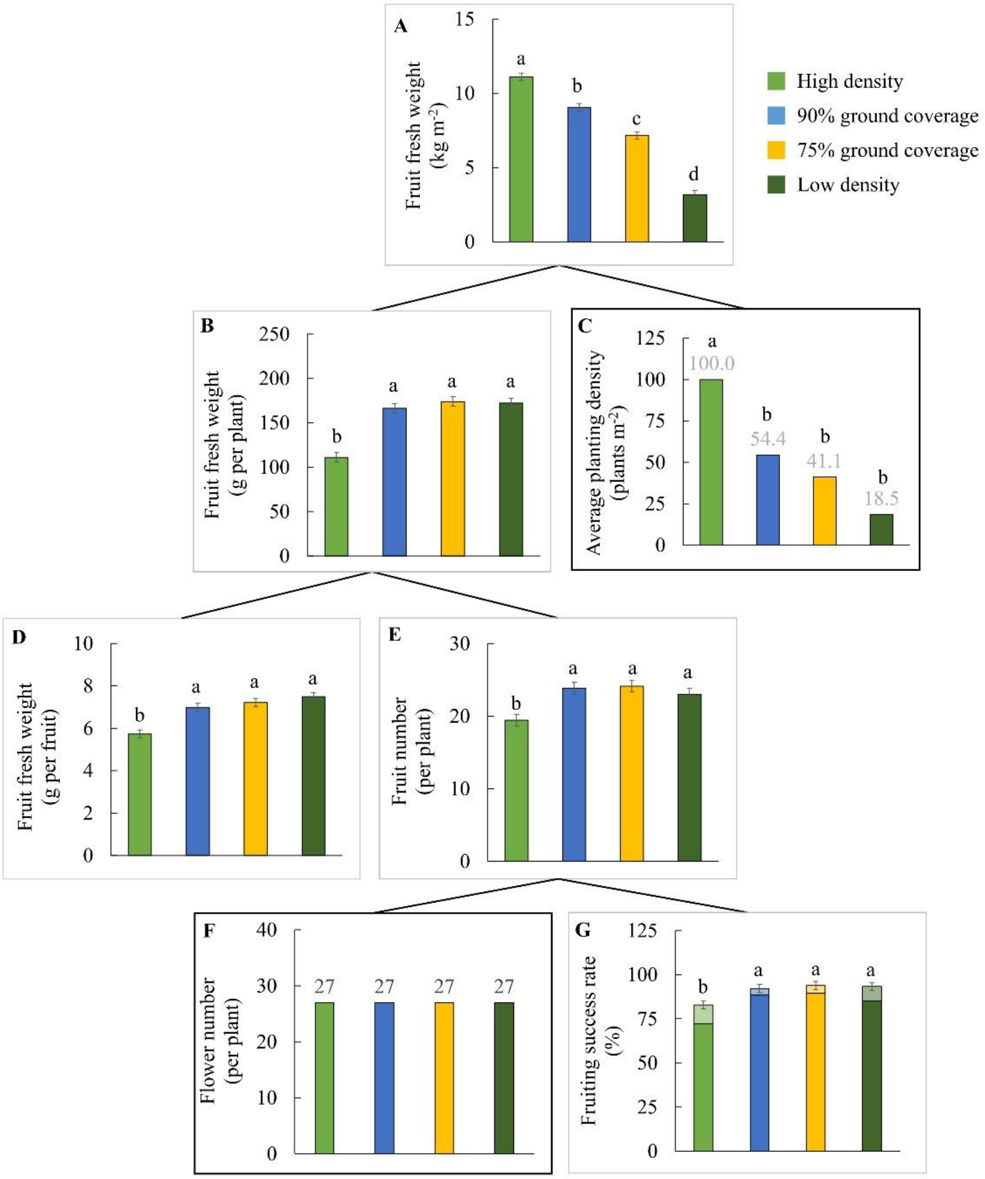

Figure S4: Effects of planting density on yield components: (A) Fruit fresh weight of red-ripe harvested fruits from 1 m^2 cultivation area over 100 days; (B) fruit fresh weight per plant; (C) average planting density; (D) calculated average fresh weight of individual fruits; (E) harvested fruit number per plant; (F) flower number per plant. Plants had been pruned to 27 flowers; and (G) flowers' fruiting success rate into harvested (full color) and not harvested (green or rotten; lighter color) fruits. The letters indicate significant differences (LSD test, $p = 0.05$). Data was the means over three blocks ($n = 3$) each with a canopy consisting of 12 replicate plants. Error bars indicate standard errors of means. The density treatments resulted in 100, 54, 41 and 19 plants per m^2 on average. GC = ground coverage.

5 Supplementary S5 – Data and figures on timing of flowering and fruit ripening

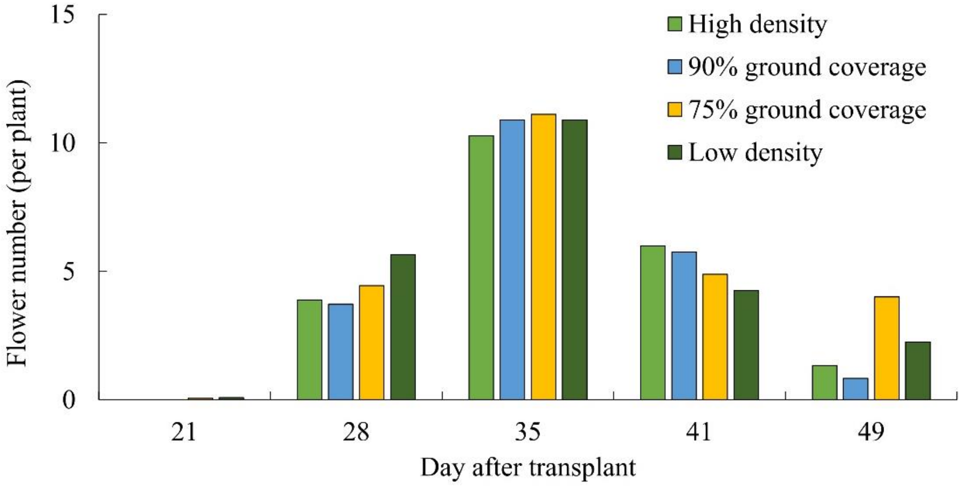

Figure S5A: Number of open flowers per plant averaged per density treatment per day after transplant.

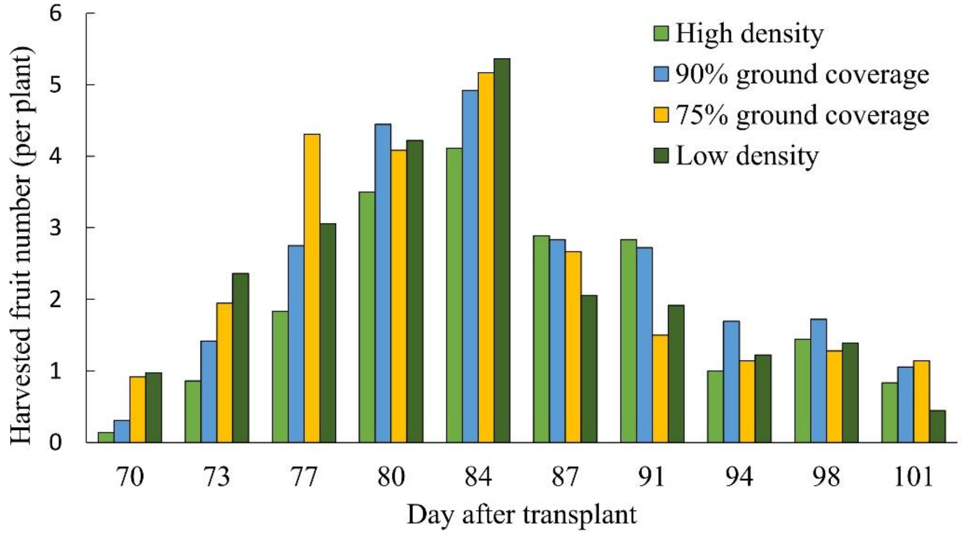

Figure S5B: Number of harvested fruits per plant averaged per density treatment (n=4) per harvest day as a function of day after transplant.

6 Supplementary S6 – Data supporting Figures 3 to 7

Table S6: Values shown in and supporting Figures 3 to 7. The letters indicate significant differences (LSD test, p = 0.05). Data is averaged per treatment (n = 4; each with a canopy consisting of 12 replicate plants) over three blocks (n = 3). The density treatments resulted in 100, 54, 41 and 19 plants per m^2 on average.

**Figure 3**		
Total (green and red-ripe) fruit fresh weight over 100 days (kg m^{-2})	Mean	LSD group
High density	12.68	a
90% ground coverage	9.42	b
75% ground coverage	7.48	c
Low density	3.25	d
Total (green and red-ripe) fruit dry weight over 100 days (g m^{-2})		
High density	927.95	a
90% ground coverage	888.88	a
75% ground coverage	751.77	b
Low density	297.49	c
Fruit fresh weight to dry weight ratio (green and red-ripe fruits)		
High density	13.66	a
90% ground coverage	10.60	b
75% ground coverage	9.95	b
Low density	10.98	b
Whole-plant dry weight on day 100 (kg m^{-2})		
High density	1.58	a
90% ground coverage	1.66	a
75% ground coverage	1.38	b
Low density	0.63	c
Dry matter partitioned to all (green and red-ripe) harvested fruits (%)		
High density	0.57	a
90% ground coverage	0.54	bc
75% ground coverage	0.55	ab
Low density	0.52	c
Light interception (mol m^{-2} d^{-1})		
High density	12.27	a
90% ground coverage	10.99	b
75% ground coverage	9.12	c
Low density	6.94	d

Whole-plant intercepted light use efficiency (g dry weight mol^{-1})		
High density	1.27	b
90% ground coverage	1.49	a
75% ground coverage	1.49	a
Low density	0.90	d
Whole-plant incident light use efficiency (g dry weight mol^{-1})		
High density	1.10	a
90% ground coverage	1.16	a
75% ground coverage	0.97	b
Low density	0.44	c
**Figure 4**		
Dry weight of stems and trusses (g per plant)	Mean	LSD group
High density	2.20	c
90% ground coverage	4.48	b
75% ground coverage	5.19	a
Low density	5.59	a
Dry weight of green leaves at destructive harvest (g per plant)		
High density	3.62	b
90% ground coverage	9.40	a
75% ground coverage	9.77	a
Low density	10.57	a
Dry weight of dropped leaves until destructive harvest (g per plant)		
High density	0.95	a
90% ground coverage	0.00	b
75% ground coverage	0.00	b
Low density	0.01	b
Dry weight of all leaves at destructive harvest (g per plant)		
High density	4.58	b
90% ground coverage	9.40	a
75% ground coverage	9.77	a
Low density	10.59	a

Dry weight of red-ripe harvested fruits (g per plant)		
High density	7.77	c
90% ground coverage	15.89	b
75% ground coverage	17.69	a
Low density	17.48	a
Dry weight of unripe harvested fruits at destructive harvest (g per plant)		
High density	1.23	a
90% ground coverage	0.64	ab
75% ground coverage	0.79	ab
Low density	0.27	b
Dry matter partitioned to stems and trusses (%)		
High density	13.89	c
90% ground coverage	14.74	bc
75% ground coverage	15.52	ab
Low density	16.46	a
Dry matter partitioned to all leaves (%)		
High density	28.97	a
90% ground coverage	30.91	a
75% ground coverage	29.22	a
Low density	31.16	a
Dry matter partitioned to red-ripe harvested fruits (%)		
High density	49.19	b
90% ground coverage	52.25	a
75% ground coverage	52.88	a
Low density	51.57	ab
Dry matter partitioned to all (red-ripe, unripe) harvested fruits (g per plant)		
High density	57.12	a
90% ground coverage	54.34	bc
75% ground coverage	55.26	ab
Low density	52.38	c
**Figure 5**		

Cumulative harvested red-ripe fruit fresh weight (kg m^{-2} over 100 days)	Mean	LSD group
High density	11.10	a
90% ground coverage	9.06	b
75% ground coverage	7.16	c
Low density	3.19	d
**Cumulative harvested red-ripe fruit fresh weight (g per plant over 100 days)**		
High density	111.02	b
90% ground coverage	166.36	a
75% ground coverage	174.01	a
Low density	172.33	a
**Total harvested red-ripe fruit number per plant after 100 days**		
High density	19.44	b
90% ground coverage	23.86	a
75% ground coverage	24.14	a
Low density	23.00	a
**Individual fruit fresh weight (g per red-ripe fruit)**		
High density	5.73	b
90% ground coverage	6.98	a
75% ground coverage	7.21	a
Low density	7.49	a
**Fruiting success rate into red-ripe and green fruits (%)**		
High density	82.82	b
90% ground coverage	92.18	a
75% ground coverage	93.83	a
Low density	93.31	a
**Fruiting success rate into red-ripe fruits (%)**		
High density	72.02	b
90% ground coverage	88.37	a
75% ground coverage	89.40	a
Low density	85.19	a

Figure 6

Incident light use efficiency (g red-ripe fruit fresh weight mol^{-1})	Mean	LSD group
High density	7.74	a
90% ground coverage	6.36	b
75% ground coverage	5.07	c
Low density	2.26	d
Intercepted light use efficiency (g red-ripe fruit fresh weight mol^{-1})		
High density	8.96	a
90% ground coverage	8.16	b
75% ground coverage	7.76	b
Low density	4.55	c
**Figure 7**		
Leaf area of green leaves after 100 DAT (cm^2 per plant)	Mean	LSD group
High density	733.00	b
90% ground coverage	1213.34	a
75% ground coverage	1209.47	a
Low density	1133.46	a
Leaf dry weight of green leaves after 100 DAT (g per plant)		
High density	3.62	b
90% ground coverage	9.40	a
75% ground coverage	9.77	a
Low density	10.57	a
Specific leaf area of green leaves after 100 DAT (cm^2 g^{-1})		
High density	202.81	a
90% ground coverage	129.07	b
75% ground coverage	123.47	bc
Low density	107.22	c
**Others**		
Annual fruit fresh weight (kg m^{-2})	Mean	LSD group
High density	40.52	a
90% ground coverage	33.05	b

75% ground coverage	26.12	c
Low density	11.64	d
Harvested fruit fresh weight per plot (12 plants) over 100 days (g)		
High density	1332.29	b
90% ground coverage	1996.25	a
75% ground coverage	2088.14	a
Low density	2067.99	a
Leaf area index (m^2 m^{-2})		
High density	6.70	a
90% ground coverage	3.94	b
75% ground coverage	2.16	c
Low density	2.09	c
